# Goethite Enhances Cr(VI) Reduction by *S. oneidensis* MR-1 under Different Conditions: Mechanistic Insights

**DOI:** 10.3390/microorganisms12040754

**Published:** 2024-04-09

**Authors:** Yu Hou, Yanhong Li, Yaru Wang, Zongqiang Zhu, Shen Tang, Jie Zhang, Qiaodong Pan, Ting Hu

**Affiliations:** 1 College of Environmental Science and Engineering, Guilin University of Technology, Guilin 541004, China; 2 Guangxi Key Laboratory of Environmental Pollution Control Theory and Technology, Guilin University of Technology, Guilin 541004, China; 3 Collaborative Innovation Center for Water Pollution Control and Water Safety in Karst Area, Guilin University of Technology, Guilin 541004, China

**Keywords:** *S. oneidensis* MR-1, Cr(VI) reduction, goethite, electron shuttle

## Abstract

Chromium (Cr) contamination, widely present in the environment, poses a significant threat to both ecology and human health. Microbial remediation technology has become a hot topic in the field of heavy metal remediation due to its advantages, such as environmental protection, low cost, and high efficiency. This paper focused on using various characterization and analysis methods to investigate the bioreduction effect and mechanism of microorganisms on Cr(VI) under various influencing factors. The main contents and conclusions were as follows: *Shewanella oneidensis* MR-1 was selected as the target strain for studying its reduction of Cr(VI) at different inoculation amounts, temperatures, pH values, time intervals, etc. The results indicated that *S. oneidensis* MR-1 exhibited an optimal reduction effect on Cr(VI) at pH 7 and a temperature of 35 °C. Additionally, electron shuttles (ESs), including humic acid (HA) and 9,10-antraquinone-2,6-disulfonate (AQDS), were introduced into the degradation system to improve the reduction efficiency of *S. oneidensis* MR-1. Upon adding goethite further, *S. oneidensis* MR-1 significantly enhanced its reducing ability by converting Fe(III) minerals to Fe(II) and reducing Cr(VI) to Cr(III) during electron transfer.

## 1. Introduction

Chromium (Cr) is a heavy metal pollutant in the environment that is widely utilized in various industrial processes such as metal processing, glass ceramic manufacturing, wood preservation, leather tanning, and dye synthesis [1,2]. In soil and water environments, Cr typically exists in two stable oxidation states: Cr(VI) and Cr(III) [3]. Among these forms, Cr(VI), being a highly toxic heavy metal with solubility and carcinogenic properties [4,5], has been extensively reported to exist in diverse environments, causing significant harm to ecological security and human health. Conversely, Cr(III) generally exhibits lower solubility [6,7,8], resulting in less environmental damage. Therefore, the conversion of Cr(VI) into Cr(III) is considered an essential step for treating environmental chromium pollution [9]. The current remediation techniques for addressing Cr(VI) contamination include adsorption [10,11,12,13], ion exchange [14,15,16,17], chemical precipitation [18,19,20,21], plant-based remediation approaches [22,23,24], and microbial restoration methods [25,26]. Among these options, microbial reduction technology is regarded as the most suitable and efficient method for restoring heavy metals that meet environmental requirements. It has been widely employed in the field of chromium restoration [27,28].

*Shewanella* species is a class of metal-reducing bacteria from outer space that sustains its growth and metabolism by utilizing organic matter as a carbon source and transferring electrons to extracellular electron acceptors [29]. Mao et al. [30] discovered that *Shewanella oneidensis* MR-1 and MR-4 have the potential to degrade sulfonamides as a bacterial resource. Xu et al. [31] demonstrated that, under anaerobic conditions, *S. oneidensis* MR-1 exhibited high efficiency in reducing azo and humic substances. Chen et al. [32] identified that *S. oneidensis* MR-1 could reduce toxic Cr(VI) to less harmful Cr(III) and domestication of this strain could enhance its reduction ability and tolerance towards Cr(VI). In addition to a direct reduction of Cr(VI), *Shewanella* can facilitate the process by assisting with Fe(II). Fe(II) is generated through the reduction of Fe(III) as an electron acceptor by *Shewanella*, thereby accelerating the processing of Cr(VI) [33]. Wielinga et al.’s research [34] revealed that the restoration degree of *Shewanella algae* BrY, a seaweed litter pollutant restorer, was significantly enhanced after introducing an iron-containing buffer solution during Cr(VI) treatment. Meng et al.’s study [35] indicated that the synergistic reduction in *S. algae* BrY on Cr (VI) was promoted by both Fe(III) minerals and electron shuttle media working together. Zhu et al. [36] observed that incorporating Fe_3_O_4_ conductive materials into the sodium alginate matrix could enhance the decolorization effect on methyl orange mediated by *S. oneidensis* MR-1.

During the degradation of Cr(VI), microorganisms rely on electron shuttles (ESs) to transfer electrons to the surface of the electron acceptor, facilitating the repair process [37]. These ESs are typically natural anthraquinone-like compounds commonly found in soil environments, such as humic acid (HA) [38,39] and 9,10-anthraquinone-2,6-disulfonate (AQDS) [40,41]. However, these ESs can be susceptible to external interference. Studies have demonstrated that iron minerals, humic acid, and anthraquinone-like compounds exhibit synergistic potential for the bioreduction of Cr(VI). Mohamed et al.’s study [42] reported a reduction rate of 65% for 1.0 mM Cr(VI) after 8 h using *S. oneidensis* MR-1 alone, whereas in the presence of goethite and humic acid, the reduction rate reached 79%. Meng et al.’s study [43] found that AQDS plus iron(III) minerals significantly enhanced the bioreduction rate of Cr(VI) compared to AQDS or iron(III) minerals alone, and the synergistic effect of AQDS and iron minerals on the bioreduction of Cr(VI) was confirmed by synergy quantitative analysis.

In summary, iron is considered an effective promoter for enhancing the reduction process and serves as a booster for *S. oneidensis* MR-1’s utilization of ESs in Cr(VI) reduction and repair. Currently, there is a dearth of comprehensive research elucidating the underlying mechanism through which *Shewanella* effectively reduces Cr(VI) in the presence of iron minerals within ESs. In this study, we assessed the reduction capacity of *S. oneidensis* MR-1 towards Cr(VI) by manipulating the inoculum size, temperature, pH, and duration. Subsequently, we investigated the underlying mechanism through which goethite enhances electron shuttling in *S. oneidensis* MR-1 to facilitate Cr(VI) reduction across multiple systems containing goethite, HA, and AQDS, aiming to establish a robust theoretical framework for the further enhancement of Cr(VI) removal.

## 2. Materials and Methods

### 2.1. Microbial Culture and Medium

The strain *S. oneidensis* MR-1 was obtained from the Marine Microbial Germplasm Collection and Management Center of China (MCCC ATCC 700550). A selected colony of *S. oneidensis* MR-1 was inoculated into LB liquid medium for culture. Subsequently, the cells were collected by centrifugation at 3500 RCF for 10 min and washed with a PBS buffer three times. Finally, the cells were resuspended in an inorganic salt solution for further use. All experiments were conducted at a temperature of 25 °C. The formulation of LB liquid medium consisted of trypsin (10.0 g·L^−1^), yeast extract (5.0 g·L^−1^), and NaCl (10.0 g·L^−1^), with pH adjusted to 7.0–7.2. The formulation of the inorganic salt solution included NH_4_Cl (1.5 g·L^−1^), NaH_2_PO_4_ (0.6 g·L^−1^), CaCl_2_ (0.01 g·L^−1^), KCl (0.1 g·L^−1^), MgCl_2_·6H_2_O (0.002 g·L^−1^), MnCl_2_·4H_2_O (0.005 g·L^−1^), and Na_2_MoO_4_·2H_2_O (0.001 g·L^−1^).

### 2.2. Material Preparation

Fe(NO_3_)_3_·9H_2_O (Xilong Science Co., Ltd., Guangzhou, China) was dissolved in ultra-pure water, stirred for 24 h (pH = 1.6), and 2.5 M potassium hydroxide solution was added until the pH of the solution reached 12. The obtained high-concentration suspension was transferred to a 60 °C oven and let stand for 5 days, followed by subsequent drying of the resulting paste at 60 °C to obtain goethite. HA and AQDS were purchased from Rin En Technology Co., Ltd. in Shanghai, China. 1,3-bis [tri (hydroxymethyl) methylamino] propane chromium solution (BTP) with ultra-pure water configuration.

### 2.3. Cr(VI) Reduction Experiments under Different Environmental Conditions

The reduction experiment of Cr(VI) was conducted under various environmental conditions to verify the ability of the MR-1 strain to reduce Cr(VI). The experimental systems included a univariate system with *S. oneidensis* MR-1, a bivariate system with *S. oneidensis* MR-1 + HA/AQDS, and a ternary system with *S. oneidensis* MR-1 + HA/AQDS + goethite. A total of 30 mM of sodium lactate and 20 mM of BTP solution were added into the anaerobic bottle, and the concentration of Cr(VI) was set to 20 mg·L^−1^. The effects of the *S. oneidensis* MR-1 inoculation amount (2%, 4%, 6%, 8%, 10%, 12%, 14%, 16%, 18%, and 20%), temperature (25 °C, 35 °C, 45 °C, and 55 °C), pH (5, 6, 7, 8, 9, and 10), and reaction time (0.5 h, 1 h, 2 h, 4 h, 6 h, 8 h, 10 h, 12 h, 16 h, 20 h, and 24 h) on the reduction effect of each system on Cr(VI) were investigated. The total reaction volume in each anaerobic bottle was fixed at 50 mL, sealed under a sterile N_2_ atmosphere, and cultured at 35 °C for 24 h in a constant-temperature shaking incubator.

### 2.4. Analysis Method of Cr(VI)

The concentration of Cr(VI) was determined by the extraction of the supernatant through a colorimetric method using a UV-5800 (PC) UV–Vis spectrophotometer by reaction with diphenylcarbazide at 540 nm [44]. The total chromium content in the solution was determined by ICP-OES (Optima 7000 DV, Perkin-Elmer, Waltham, MA, USA). The chromium reduction rate is expressed as C_t_/C_0_. Where C_0_ is the concentration of Cr(VI) before reduction, C_t_ is the concentration of Cr(VI) after reduction.

### 2.5. Characterization Analysis Method

We collected samples related to the reduction products from *S. oneidensis* MR-1 cells. After centrifugation at 8000 rpm for 15 min, they were washed three times with deionized water and freeze-dried for further analysis. We used X-ray diffraction (XRD, X’Pert PRO, Panace, Almelo, Netherlands) to study crystal phase structure changes before and after Cr(VI) reduction. The morphology and elemental composition of the cell surface were characterized using a scanning electron micrography–energy spectrometer (SEM-EDS, Hitachi SU8010, HITACHI, Tokyo, Japan) and field emission scanning electron microscope (JSM-7900F field emission scanning electron microscope, JEOL, Tokyo, Japan). The elemental valence changes on the sample surface before and after Cr(VI) reduction were analyzed by X-ray photoelectron spectroscopy (XPS, ESCALAB 250Xi, Thermo Fisher, Waltham, MA, USA). The surface functional groups of *S. oneidensis* MR-1 before and after Cr(VI) reduction were studied by Fourier transform infrared spectroscopy (FTIR, Nicolet Nexus-6700, Thermo Nicolet Co., Waltham, MA, USA). The Origin Lab 2021 software (Origin Lab, Northampton, MA, USA) was used for data processing.

### 2.6. Statistical Analysis

All experiments were performed in triplicate, and the results were presented as the mean ± standard deviation. Data processing was conducted using IBM SPSS Statistics 26 software (IBM SPSS, Armonk, NY, USA). Line and bar graphs were generated using Origin Lab 2021 software (Origin Lab, Northampton, MA, USA).

## 3. Results and Discussion

### 3.1. Influence of Different Environmental Factors on the Reduction Effect of Cr(VI)

**Figure 1:**
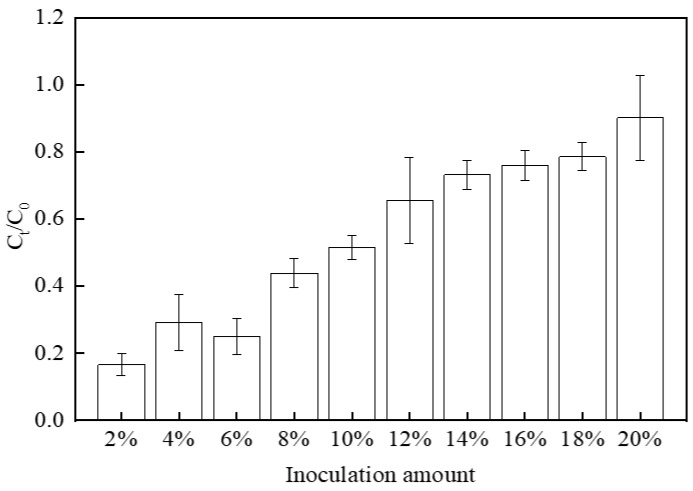
Effect of *S. oneidensis* MR-1 inoculum on Cr(VI) reduction.

The inoculation amount of *S. oneidensis* MR-1 directly influences the reduction process of Cr(VI). In relative terms, a higher concentration of *S. oneidensis* MR-1 promotes the reduction process of Cr(VI). Figure 1 illustrates that when the inoculation amount is low (<10%), its impact on Cr(VI) reduction is limited. However, as the concentration exceeded 10%, an increase in *S. oneidensis* MR-1 led to a gradual enhancement in its ability to reduce Cr(VI). Notably, when increasing the inoculation amount from 18% to 20%, a significant improvement in the Cr(VI) reduction rate was observed for 20% *S. oneidensis* MR-1 (referred to as M2), which served as our research object while using 10% *S. oneidensis* MR-1 as our benchmark group (referred to as M1) for a clearer demonstration.

**Figure 2:**
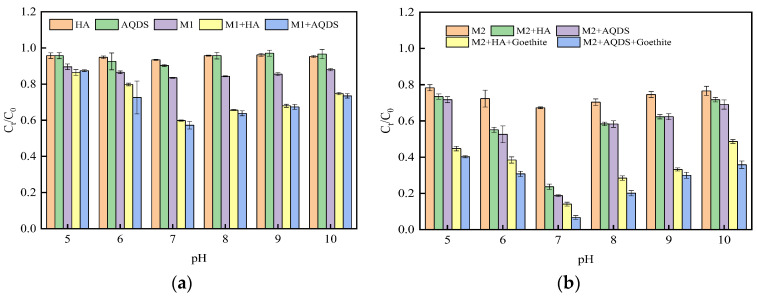
Effect of reaction system pH on Cr(VI) reduction by *S. oneidensis* MR-1 ((**a**) System system at M1 concentration; (**b**) System system at M2 concentration).

In the process of Cr(VI) reduction by *S. oneidensis* MR-1, the change in ion morphology at the active site of Cr(VI) reductase is a crucial factor influencing its reduction efficiency [45]. As depicted in Figure 2, within the pH range of 5 to 10, when the inoculum amount of *S. oneidensis* MR-1 was 10%, both binary systems composed of *S. oneidensis* MR-1 and HA or *S. oneidensis* MR-1 and AQDS exhibited higher intensity in reducing Cr(VI) compared to a single strain; similarly, a trend was also observed when the inoculum amount of *S. oneidensis* MR-1 was 20%. Figure 2a demonstrates that as the pH increases to 7, both single-strain systems and binary systems reach their maximum intensity in reducing Cr(VI). This phenomenon could be attributed to an increase in the number of active sites for Cr(VI) reductase under neutral conditions, thereby promoting the overall reduction process. Figure 2b reveals that under M2 conditions, *S. oneidensis* exhibited a stronger ability to reduce Cr(VI) compared with M1 conditions alone. Furthermore, under M2 conditions, the binary system showed more efficient reduction effects on Cr(VI) than a single strain did alone. The introduction of goethite into the system enabled *S. oneidensis* to achieve its maximum degradation capability towards Cr(VI). Therefore, adding ESs enhanced the reduction strength of *S. oneidensis* towards Cr (VI), while incorporating goethite further amplified it.

**Figure 3:**
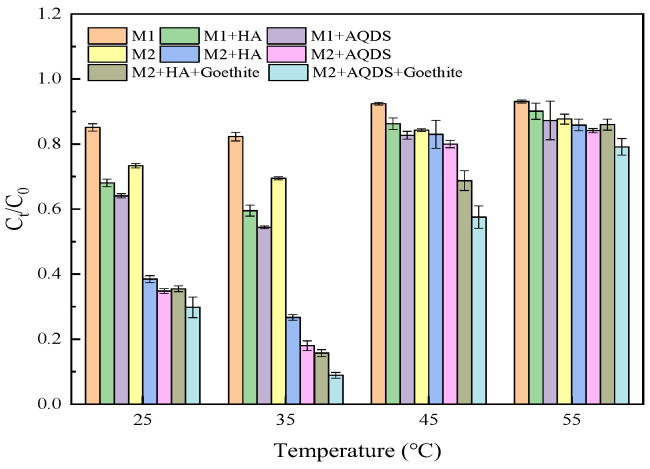
Effect of reaction system temperature on Cr(VI) reduction by *S. oneidensis* MR-1.

The reaction temperature exerted a significant influence on the microbial growth activity and reduction rate. As depicted in Figure 3, the reduction intensity of *S. oneidensis* towards Cr(VI) exhibited a notable increase as the temperature rose from 25 °C to 35 °C. However, once the temperature surpassed 35 °C, the reduction ability of *S. oneidensis* towards Cr(VI) started to decline. At 35 °C, each system demonstrated optimal Cr(VI) reduction efficacy, indicating its suitability for reduction purposes. Similar to pH, microorganisms also exhibited specific requirements for growth temperature. When the temperature was below 35 °C, microbial activity was constrained and failed to fully activate the sites responsible for Cr(VI) reductase activity; when it exceeded 35 °C, it hindered protein function, nucleic acids’ performance, and cellular components in *S. oneidensis* MR-1, leading to a decrease in reduction effectiveness. This pattern was observed consistently across different systems under both M1 and M2 conditions, with the ternary system exhibiting superior Cr(VI) reduction intensity.

**Figure 4:**
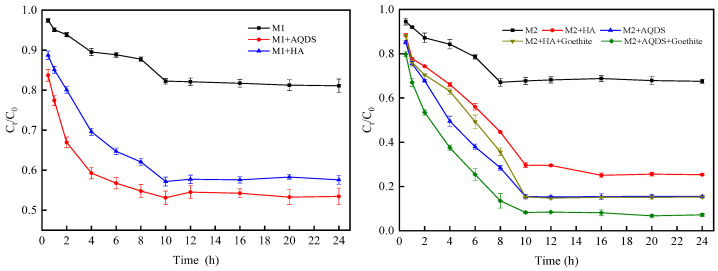
Effect of reaction time on Cr(VI) reduction by *S. oneidensis* MR-1.

The reduction time had a significant impact on the reduction of Cr(VI) by *S. oneidensis* MR-1. As depicted in Figure 4, over a period of 10 h, the rates of Cr(VI) reduction gradually increased in all three systems and reached equilibrium after 10 h. This phenomenon could be attributed to the maximum activity exhibited by *S. oneidensis* MR-1 during the initial stage, leading to its gradual decrease and eventual equilibrium due to reaching its maximum capacity for reducing Cr(VI). Notably, compared to the other two systems, *S. oneidensis* MR-1 demonstrated a superior ability to reduce Cr(VI) within the ternary system. There was minimal disparity observed in the equilibrium time required for Cr(VI) reduction between both binary and ternary systems when compared with single *S. oneidensis* MR-1 alone. These findings suggested that an electron shuttle aids in maximizing the activity of *S. oneidensis* MR-1, while goethite further enhances its capability for reducing Cr(VI), albeit without shortening the overall reduction time.

By fitting the reduction process with a quasi-first-order kinetic equation, the reduction rate values and trends of each reaction system can be more intuitively observed. For the fitting of the quasi-first-order kinetic model of the reduction process, the rate can be calculated and expressed by a quasi-first-order kinetic equation.

*Ln* (*q_e_* − *q_t_*) = *Ln q_e_* − *K t*, (1)

*q_t_* = (*C*_0_ − *C_t_*) × *V/m*, (2)

*q_e_* = (*C*_0_ − *C_e_*) × *V/m*, (3)

In this equation, *q_e_* represents the equilibrium reduction amount in mg·g^−1^; *q_t_* represents the reduction amount at any given time in mg·g^−1^; *K* represents the quasi-first-order rate constant for reduction in min^−1^; *C*_0_ (mg/L) is the initial concentration of Cr(VI); *C_t_* (mg/L) and *C_e_* (mg/L) are the concentrations of Cr(VI) at the reaction time (*t*) and the equilibrium time, respectively; *V* (L) is the total volume of the solution; and *m* (g) is the dosage of the reactants.

**Figure 5:**
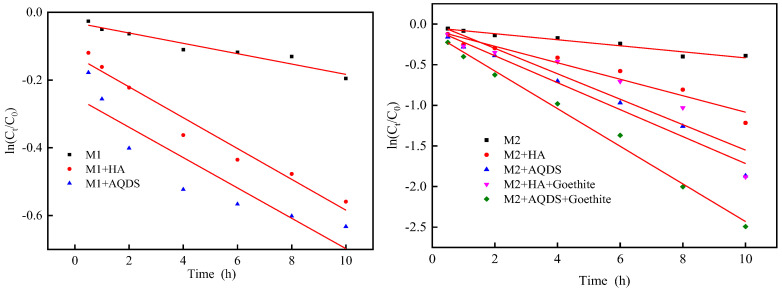
Pseudo-first-order kinetics fitting pattern of *S. oneidensis* MR-1 reduction of Cr(VI).

The quasi-first-order kinetic fitting plot of Cr(VI) was generated with time as the x-axis and ln(*q_e_* − *q_t_*) as the y-axis (Figure 5).The fitted data were analyzed, and the corresponding parameters are listed in Table 1 and Table 2. To quantify the promotion effect of Ess or goethite on Cr(VI) reduction process, the enhancement factor (*EF*) was introduced, which can be calculated using the following method:

*EF* = *K_i_*/*K*_0_, (4)

In the formula, *EF* represents the enhancement coefficient of electron shuttle or goethite for *S. oneidensis* MR-1. When *EF* is greater than 1, there is an enhanced reduction effect; when *EF* is less than 1, there is no enhanced reduction effect. *K*_0_ represents the quasi-first-order kinetic constant of *S. oneidensis* MR-1 reducing Cr(VI); *K_i_* represents the quasi-first-order kinetic constant of *S. oneidensis* MR-1 reducing Cr(VI) in the presence of an electron shuttle or goethite.

Based on the parameters provided in Table 1 and Table 2, the individual strain *S. oneidensis* MR-1 exhibited reduction rates of 0.0153 and 0.0372 for Cr(VI), indicating an enhanced capacity to reduce Cr(VI) with increasing inoculum size (M2 > M1), which correlated with the reaction rate and enhancement coefficient relationship. These findings were consistent with the results obtained from both the experiment investigating the effect of inoculum size and the kinetics study on reduction. Under M2 conditions, in the presence of Ess (HA, AQDS), it effectively reduced Cr(VI) compared to using only *S. oneidensis* MR-1 based on their correlation between reaction rates and enhancement coefficients. Introducing goethite further increases the reduction rate of Cr(VI) in a ternary system compared to a binary system and exhibits an almost twofold greater enhancement effect than before. It is confirmed that iron minerals promote the reduction of Cr(VI) by *S. oneidensis* MR-1 in the presence of Ess [41].

**Figure 6:**
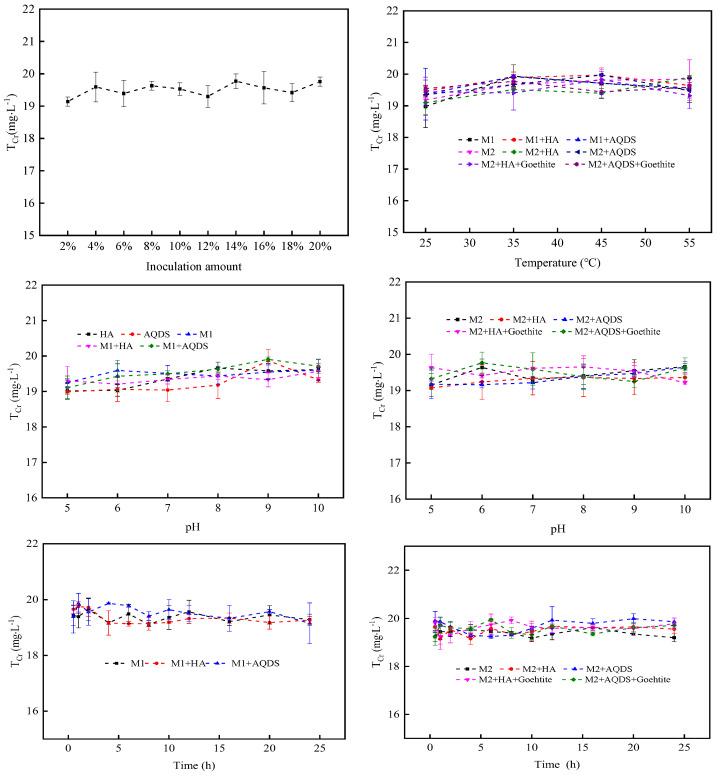
Changes in total Cr in the reaction system.

As depicted in Figure 6, the total Cr content in various systems exhibited minimal variation under different reaction conditions. Regardless of changes in pH, temperature, or time, the overall concentration of chromium remains relatively stable within the range of 19–20 mg·L^−1^, displaying negligible fluctuations compared to the initial concentration of 20 mg·L^−1^. This observation suggested a high degree of stability regarding the total chromium concentration within the reaction system. However, a slight disparity existed between the total chromium concentration and that of Cr(VI) at the beginning due to adsorption phenomena occurring on both *S. oneidensis* MR-1 and material surfaces.

### 3.2. Mechanism Analysis of Cr(VI) Reduction Process

#### 3.2.1. SEM and EDS Analysis before and after Reduction

**Figure 7:**
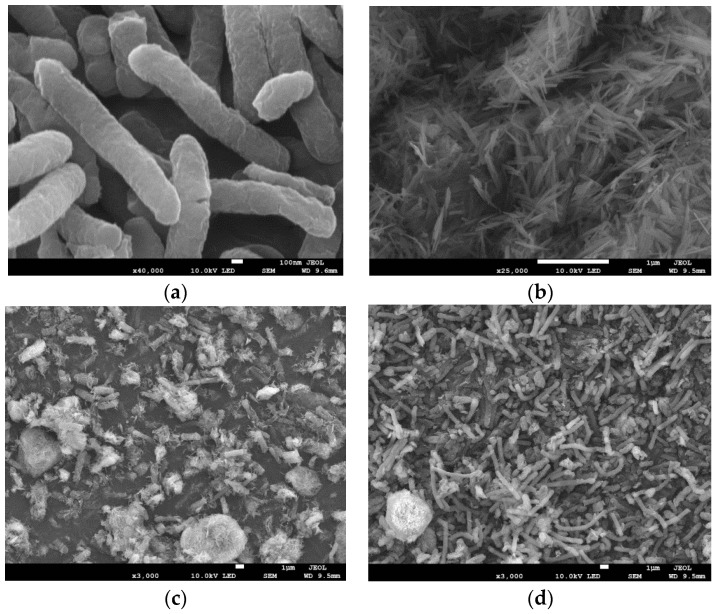
Electron microscope images of *S. oneidensis* MR-1 and goethite and their composite reduction of Cr(VI) ((**a**) *S. oneidensis* MR-1; (**b**) goethite; (**c**) *S. oneidensis* MR-1, AQDS, and goethite adsorbed chromium; (**d**) *S. oneidensis* MR-1, HA, and goethite adsorbed chromium).

The electron microscopy images of *S. oneidensis* MR-1 (a), goethite (b), *S. oneidensis* MR-1, AQDS, and goethite (c), and *S. oneidensis* MR-1, HA, and goethite (d) after Cr(VI) treatment are presented in Figure 7. Figure 7a shows the complete topography of *S. oneidensis* MR-1, revealing intact rod-and-stick structures of the bacteria cells cultured at 30 °C for 24 h without any damage observed. Figure 7b displays the electron microscopy image of goethite, exhibiting regular strips that are consistent with previous characterization findings by Russell et al. [46]. By observing Figure 7c,d, it can be noted that in the presence of an electron shuttle (AQDS or HA), when *S. oneidensis* MR-1 reduces Cr(VI), goethite adheres to its surface without causing significant damage to its overall structure, indicating its participation in the reduction process as a non-damaging component.

**Figure 8:**
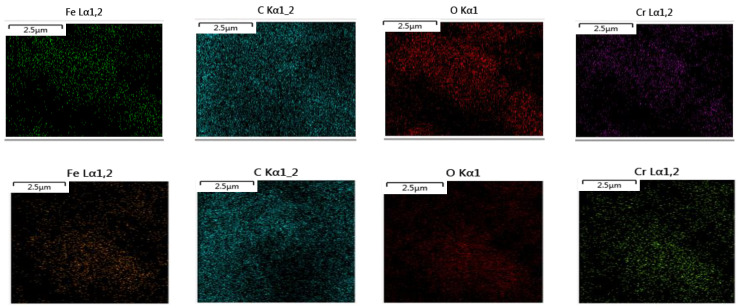
EDS spectrum of *S. oneidensis* MR-1, HA/AQDS, and goethite after Cr(VI) reduction.

SEM-EDS analysis enables the observation of morphological characteristics as well as the determination of element composition and its distribution on material surfaces. The EDS spectrum of *S. oneidensis* MR-1, HA/AQDS, and goethite after Cr(VI) reduction is presented in Figure 8, revealing a uniform distribution of carbon (C), oxygen (O), iron (Fe), and other elements on the sample surface. This confirms the successful dispersion of synthesized goethite onto the material surface. Furthermore, chromium distribution mapping demonstrates an even distribution on the material surface during Cr(VI) reduction by *S. oneidensis* MR-1, HA/AQDS, and goethite.

#### 3.2.2. XRD Analysis before and after Reduction

**Figure 9:**
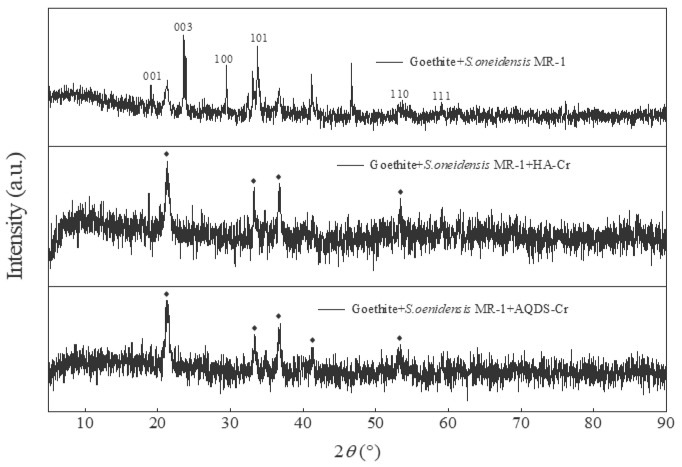
XRD of *S. oneidensis* MR-1 and goethite and *S. oneidensis* MR-1, HA/AQDS, and goethite after Cr(VI) reduction.

XRD analysis was conducted to investigate the crystal phase structure of samples before and after Cr(VI) reduction. The samples of *S. oneidensis* MR-1 and goethite and *S. oneidensis* MR-1, HA/AQDS, and goethite were subjected to XRD analysis after Cr(VI) treatment, and the obtained results were compared with standard cards for identification purposes. As shown in Figure 9, the diffraction peaks observed at 2θ = 19.24°, 23.60°, 29.56°, 53.55°, and 59.34° corresponded to (001), (003), (100), (101), (110), and (111) in PDF 81-0643, respectively, indicating the presence of high-quality goethite crystals in the prepared material. By analyzing the XRD patterns of *S. oneidensis* MR-1, HA/AQDS, and goethite after Cr(VI) treatment, it was observed that the intensity of diffraction peaks corresponding to planes 001 and 100 increased, while those corresponding to planes 003, 101, and 111 decreased significantly due to the Cr(VI) reduction process affecting the crystal structure of goethite. The involvement of acicular goethite in the Cr(VI) reduction reaction on the surface of *S. oneidensis* MR-1 was confirmed through a combination of SEM and EDS analyses.

#### 3.2.3. FTIR Analysis before and after Reduction

**Figure 10:**
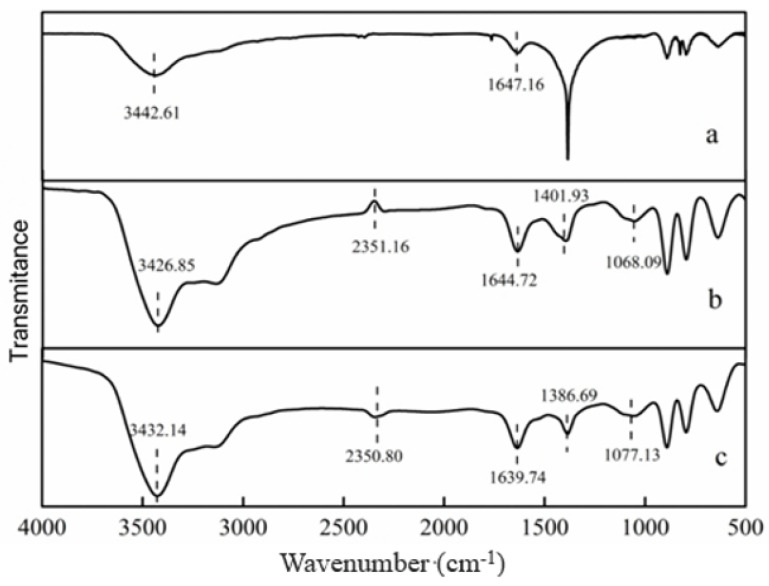
Fourier transform infrared spectrum ((**a**) *S. oneidensis* MR-1 and goethite; (**b**) *S. oneidensis* MR-1, HA, and goethite after Cr(VI) reduction; (**c**) *S. oneidensis* MR-1, AQDS, and goethite after Cr(VI) reduction).

The efficacy of Cr(VI) reduction by *S. oneidensis* MR-1, HA/AQDS, and goethite primarily relied on the specific types of functional groups involved. The FTIR spectrum was employed to analyze the alterations in functional groups pre- and post-reduction of Cr(VI) by *S. oneidensis* MR-1 and goethite. As depicted in Figure 10, the absorption band around 3400 cm^−1^ corresponded to H-O bond vibrations, while the absorption band near 1640 cm^−1^ indicated C=O vibrations. Furthermore, the peak at approximately 1386 cm^−1^ represented bending vibrations of carboxyl and phenol groups as well as O-H in-plane deformation vibrations, whereas the absorption band at 1400 cm^−1^ denoted C-O vibrations [37,47]. Previous studies have demonstrated that phenol groups serve as key functional groups that are responsible for reducing Cr(VI) to Cr(III), ultimately forming carbonyl/carboxylic groups. Additionally, carboxyl groups act as binding sites for stabilizing Cr(III) [48], suggesting that the presence of goethite generates key functional groups during the reduction of Cr by *S. oneidensis* MR-1, thereby improving the efficiency of the system.

#### 3.2.4. XPS Analysis before and after Reduction

**Figure 11:**
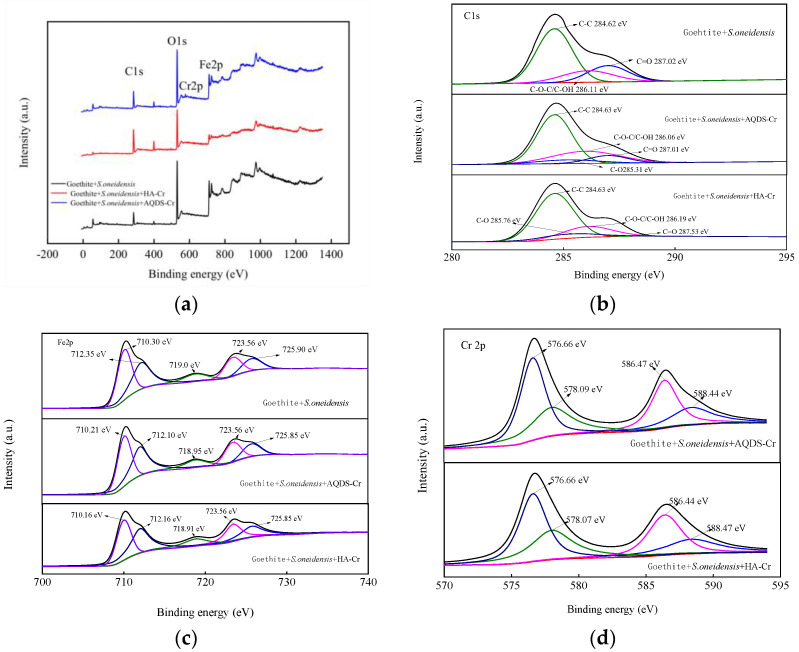
XPS spectra of goethite composite *S. oneidensis* MR-1 before and after Cr(VI) reduction ((**a**) Peak of goethite composite MR-1 before and after Cr(VI) reduction; (**b**) Spectra of C 1s before and after reduction; (**c**) Spectra of Fe 2p before and after reduction; (**d**) Spectra of Cr 2p 1s before and after reduction).

The changes in the valence states of major elements in different systems were investigated by analyzing the XPS spectra before and after reduction, and the results are shown in Figure 11. Prior to Cr(VI) reduction, the *S. oneidensis* MR-1 and goethite samples primarily consisted of C, O, and Fe. However, following Cr(VI) reduction, the XPS spectra of *S. oneidensis* MR-1, HA/AQDS, and goethite samples exhibited a prominent chromium (Cr) peak. The Fe 2p and C 1s spectra are presented in Figure 11a–c, respectively. It is noteworthy that both before and after reduction, the goethite and *S. oneidensis* MR-1 samples contained oxygen-containing functional groups such as C-O-C/C-OH and C=O, which played a crucial role in the Cr(VI) reduction process. For a more detailed analysis of the Fe 2p photoelectron peak, please refer to Figure 11c: specifically, the range of 710–712.3 eV corresponds to Fe 2p1/2, while the range of 723–726 eV corresponds to Fe 2p3/2 [49]. Notably, peaks at energies of approximately 725.9 eV, 712.10 eV, and 718.90 eV indicated Fe(III), whereas peaks at energies around 723.56 eV and 710.21 eV represented Fe(II). Regarding chromium species identification, the energy range between 576 and 578 eV is associated with the Cr 2p3/2 orbital, and the range between 586 and 589 eV corresponds to Cr [50,51]. As shown in Figure 11d, the peaks at 588.44 eV and 578.07 eV are related to Cr(VI) compounds, while the peaks at 576.66 eV and 586.47 eV are related to Cr(III) compounds. This result was roughly consistent with the above Fe 2p analysis results. The research findings demonstrated that *S. oneidensis* MR-1 effectively enhanced the surface availability of reduction sites through Fe(III) reduction to Fe(II). In conjunction with the total chromium analysis, we observed rapid transfer to Cr(VI) leading to its reduction to Cr(III), which significantly contributes to the enhanced removal efficiency of Cr(VI). This conclusion was derived from a comprehensive analysis of reduction kinetics.

## 4. Conclusions

The present study provides experimental evidence that goethite facilitates the reduction of Cr(VI) by *S. oneidensis* MR-1 in the presence of ESs. The reduction effect of *S. oneidensis* MR-1 on Cr(VI) was analyzed under different conditions, and it was found that the optimal reduction effect occurred at 20% inoculation, a 35 °C temperature, and pH 7. The ternary system exhibited a stronger ability to reduce Cr(VI) compared to the binary and unitary systems, indicating that goethite addition facilitated the reaction. Kinetic analysis confirmed that the presence of Fe(III) promoted Cr(VI) reduction. Combined with SEM, EDS, XRD, FTIR, and XPS analysis results, it was verified that goethite was reduced to Fe(II) by *S. oneidensis* MR-1, thereby significantly enhancing the strain’s reduction reaction towards surface Cr(VI) and facilitating its conversion into Cr(III). In summary, in the presence of an electron shuttle, *S. oneidensis* MR-1 benefited from reducing Cr(VI), while introducing goethite increased Fe(III), which is subsequently reduced to Fe(II) by *S. oneidensis* MR-1, thus increasing the active sites and promoting Cr(VI) reduction.

This study provides novel insights into the application of goethite and ESs for bioremediation in Cr(VI)-contaminated areas, establishing a theoretical foundation for *S. oneidensis* MR-1 microorganisms enhancing Cr(VI) removal. Furthermore, the proposed method offers a promising bioremediation technique for Cr(VI)-contaminated soil (including wastewater) in the natural environment. For instance, the introduction of electron shuttles and dissimilatory iron-reducing bacteria such as *Shewanella* can significantly enhance the bioreduction process of Cr(VI) at chromium-contaminated sites that are rich in iron. However, it is essential to further investigate the physiological and biochemical reactions and gene expression of *Shewanella* during Cr(VI) reduction to gain a comprehensive understanding of the microbial reduction mechanism. Additionally, the experiment was conducted under rigorous laboratory conditions. Further studies that extrapolate these findings to natural environments will offer even more valuable insights.

## Figures and Tables

**Table 1:** M1 system reduction Cr(VI) fitting kinetic equation-related parameters.

System of Reaction	*K*/h^−1^	R^2^	*EF*
M1	0.0153 ± 0.0017	0.9280	/
M1 and HA	0.0402 ± 0.0038	0.9589	2.63
M1 and AQDS	0.0455 ± 0.0086	0.9183	2.97

**Table 2:** M2 system reduction Cr(VI) fitting kinetic equation-related parameters.

System of Reaction	*K*/h^−1^	R^2^	*EF*
M2	0.0372 ± 0.0013	0.9429	/
M2 and HA	0.1014 ± 0.0105	0.9389	2.72
M2 and AQDS	0.1659 ± 0.0012	0.9731	4.43
M2, HA, and goethite	0.1566 ± 0.0249	0.8656	4.21
M2, AQDS, and goethite	0.2317 ± 0.0090	0.9911	6.23

## Data Availability

Data are contained within the article.
